# Real-Life Early Anthropometric, Lipid and Liver Changes after Direct-Acting Antiviral Therapy in PLWHIV with HCV Co-Infection

**DOI:** 10.3390/jcm11092639

**Published:** 2022-05-07

**Authors:** Sergio Ferra-Murcia, Antonio Ramón Collado-Romacho, Bruno José Nievas-Soriano, Fernando Reche-Lorite, Tesifón Parrón-Carreño

**Affiliations:** 1 Infectious Diseases Unit, Internal Medicine Service, Torrecárdenas University Hospital, 04009 Almeria, Spain; sergio.ferra.sspa@juntadeandalucia.es (S.F.-M.); antonior.collado.sspa@juntadeandalucia.es (A.R.C.-R.); 2 Department of Nursing, Physiotherapy and Medicine, University of Almeria, 04120 Almeria, Spain; tpc468@ual.es; 3 Department of Mathematics-Statistics and Operations Research Area, University of Almeria, 04120 Almeria, Spain; freche@ual.es

**Keywords:** HCV/HIV co-infected, direct-acting antivirals, metabolic impact, comorbidity PLWHIV, before-after DAA therapy

## Abstract

Treatment with interferon-free direct-acting antivirals (DAA) has become the gold standard in chronic hepatitis C virus (HCV) infection. Nevertheless, little research about the metabolic impact of achieving sustained virological response (SVR) is available in HCV/HIV co-infected patients. This research aimed to evaluate early anthropometric, lipid and liver parameters changes after achieving SVR 12 weeks after treatment (SVR12). A real-life retrospective descriptive before-after study assessed 128 DAA treatment episodes from 2015 to 2019 in HCV/HIV co-infected patients. Anthropometric parameters (weight, body mass index), lipid profile, genotype (GT) and viral load, liver data (basics laboratory necroinflammatory parameters and transient elastography (TE)) were collected before treatment with DAA (baseline), and when SVR12 was achieved. Significant increases (*p* < 0.01) were found in the early lipid profile, measured by LDLc (84.6 ± 35.0 vs. 108.6 ± 35.1 mg/dL) and total cholesterol (161.3 ± 41.0 vs. 183.3 ± 41.6 mg/dL). Significant changes (*p* < 0.05) were found in liver parameters, measured by ALT (58.2 ± 34.0 vs. 22.0 ± 16.0 U/L), bilirubin (0.8 ± 0.6 vs. 0.6 ± 0.5 mg/dL), albumin (4.2 ± 0.4 vs. 4.3 ± 0.3 g/dL) and liver stiffness (LS) (13.7 ± 13.3 vs. 11.8 ± 12.1 kPa). The main conclusions were that the use of DAA has an early negative impact on lipid metabolism. Achieving SVR12 against HCV leads to an early improvement in liver function and LS in HCV/HIV co-infected patients without interference with antiretroviral treatment (ART) and DAA. Short-term close lipid monitoring may be necessary when combining protease inhibitors. HCV-GT-3/HIV co-infected patients might require further close monitoring for residual fibrosis. These findings can be relevant for actual clinical practice.

## 1. Introduction

Hepatitis C virus (HCV) infection is a ubiquitous disease with a variable prevalence [1]. In the last 20 years, sexual transmission has acquired greater relevance, as rectal elimination of HCV in infected individuals and specific practices in sexual contexts, such as the intentional sex under the influence of psychoactive drugs, so-called «Chemsex», may lead to an increased risk of transmission [2,3,4,5]. It is estimated that 400,000 people globally will die annually from end-stage liver disease. Around 70% of persons infected with HCV will develop a chronic infection with a risk of progression to cirrhosis ranging from 15% to 30% over 20 years [1]. The consequences of chronic HCV infection in patients living with the human immunodeficiency virus (PLWHIV) are significant. They may present accelerated progression, higher rates of end-stage liver disease, and lower survival after an episode of liver decompensation, especially in those with an advanced degree of immunosuppression [6].

The HCV elimination in PLWHIV reduces necroinflammation and the incidence of hepatocellular carcinoma. In addition, some authors have described a reduction in the risk of antiretroviral drug-induced liver damage [7,8,9]. Chronic HCV co-infection in PLWHIV had lower response rates to treatment with pegylated interferon (PEG) and Ribavirin (RBV) when compared to HCV mono-infected patients, which can be different according to the HCV genotype (GT) and individual characteristics [10]. However, interferon-free DAA therapy has shown high efficacy rates in HCV infection in PLWHIV, thanks to the development of well-tolerated DAA oral regimens with a better adherence in this sub-group of HCV/HIV co-infected patients, increasing response rates such as HCV mono-infected patients [11]. The combined management of ART and DAA poses a significant clinical challenge when treating HCV/HIV co-infected patients in real-life [12].

Recent articles such as Iossa D et al. [13] highlight the development of a proatherogenic pattern after HCV eradication in mono-infected patients treated with DAA. Other works [14,15,16] show how HCV competes with the hepatic low-density lipoprotein cholesterol (LDLc) receptor, leading to hypobetalipoproteinemia. It allows serum lipid particles in the HCV-infected patient to interfere with circulation, enter the hepatocyte, and facilitate viral replication [14]. Another facilitating agent to the entrance of HCV into the hepatocyte is related to the functionality of low-density lipoproteins, called “lipoviro-particles” (LVP), with which HCV is associated [15]. The influence of HCV on lipid metabolism is based on three essential mechanisms: increasing lipogenesis, reducing lipid secretion, and decreasing their degradation [16]. Due to these interactions, chronic HCV infection is associated with low serum ApoB, LDLc, and total cholesterol levels [17].

Sustained virological response 12 (SVR12) is defined by undetectable HCV-RNA in serum or plasma 12 weeks after the end of therapy (EOT). It is considered a universal criterion to define HCV cure both in mono-infected (HCV) and co-infected patients (HCV/HIV) [18,19]. Several studies have based the analysis on the impact on HCV eradication in mono-infected patients and the rapid and durable persistence in the regression of liver stiffness (LS) in HCV/HIV co-infected patients after being treated with PEG/RBV. However, limited data are available regarding the early metabolic impact after HCV eradication in PLWHIV treated with DAA [20,21,22,23,24]. In this context, we proposed to analyze a real-life cohort of PLWHIV with HCV co-infection, treated for HCV with interferon-free DAA therapy and on antiretroviral therapy (ART). Therefore, the main aim of this research was to assess the early impact of achieving SVR12 in terms of anthropometric and lipid changes after DAA therapy. We also sought to assess whether, in the short-term, significant differences between baseline vs. SVR12 were obtained in liver data, measured by basic laboratory parameters and LS by transient elastography (TE).

## 2. Materials and Methods

### 2.1. Study Design

**Figure 1:**
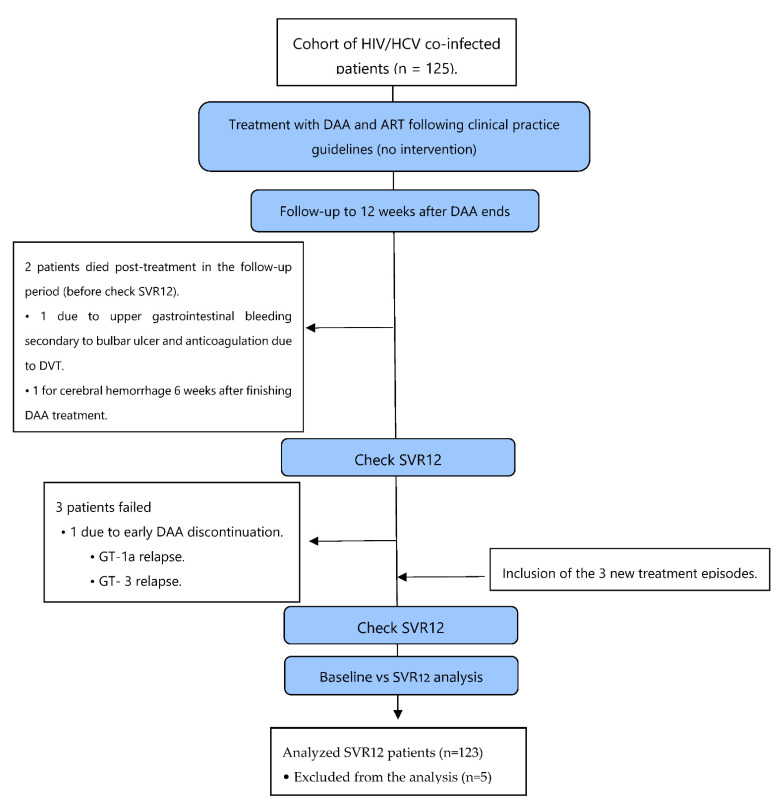
Study flowchart. DVT, deep vein thrombosis; DAA, direct-acting antiviral; ART, antiretroviral treatment; baseline, before DAA treatment; SVR12, sustained viral response 12 weeks after DAA ends; GT, HCV genotype.

An observational-descriptive before-after study was designed based on data from routine clinical practice (real-life) and retrospective analysis, as shown in Figure 1.

### 2.2. Patient Recruitment

The sample was obtained from all PLWHIV with chronic HCV co-infection under regular follow-up at the Infectious Diseases Unit of the Torrecárdenas University Hospital, the reference hospital of Almeria, Spain. Those who had received DAA therapy in interferon-free regimens from January 2015 to December 2019 were assessed. A total of 128 treatment episodes corresponding to 125 patients were evaluated, but only 123 patients who achieved SVR12 were analyzed (n = 123). All of the patients included were PLWHIV under ART and with HCV co-infection.

The ART and DAA regimen were selected following clinical practice guidelines in real life and under the criteria of the responsible physicians without interfering with the study objectives. The characteristics analyzed at the visit before DAA treatment were considered for baseline determination. The comparative before-after retrospective analysis was performed when each patient achieved SVR 12 weeks after the end of treatment (SVR12), comparing baseline versus after reaching SVR12.

### 2.3. Clinical Data Assessment

Clinical data were collected from routine medical records. The following parameters were included: demographic and anthropometric (weight, body mass index, date of birth, age at the start of DAA treatment, sex, weight), lipid profile (low-density proteins (LDLc), total cholesterol), GT and viral load (HIV and HCV), liver data (basics laboratory parameters: alanine aminotransferase (ALT), Child-Turcotte-Pugh (CTP) score, bilirubin, albumin and liver stiffness (LS) measured in kilopascals (kPa)). All variables (except demographic variables) have been collected at baseline and after SVR12.

LS was measured by transient elastography (TE): exploration was performed by one experienced operator with an ultrasound transducer placed in one of the right intercostal spaces. This allowed for the visualization of an ultrasound window with liver parenchyma characteristics. An elastic wave was emitted thanks to the probe vibration. The LS calculation measured in kPa was obtained using the propagation speed wave through the hepatic parenchyma [25]. Metavir fibrosis score was applied to estimate the degree of fibrosis with the following TE cut-off values: F0–F1: ≤6 kPa, F2: 6.1–9.4 kPa, F3: 9.5–14.5 kPa, F4: ≥14.6 kPa [26].

A secure and irreversible anonymization procedure guaranteed that the information handled did not contain personal data, following EU Regulation 2016/679 and the Spanish Organic Law 3/2018 of December 5, Protection of Personal Data and Guarantee of Digital Rights. The study complied with the ethical principles included in the Declaration of Helsinki and was approved by the Research Ethics Committee of the Province of Almeria, Spain, with reference number CI-14/2021.

### 2.4. Statistics

Analyses were performed using the statistical program SPSS v21.0 (IBM, Chicago, IL, USA) and R software for statistical analysis and graphics [27]. The quantitative variables were expressed as means accompanied by their standard deviations (mean ± SD). After applying the Shapiro-Wilk test and assessing that the sample did not follow a normal distribution, non-parametric tests were applied: before-after Wilcoxon test and a Box-and-whisker plot. A contingency table with a chi-square statistic has been created to confirm that ART and DAA’s actual use has not been distributed independently. A procedure of ranges and pseudo-range (modified two-way ANOVA) was performed with the rankFD R package since the assumptions that allow the application of the classic ANOVA were not met, being a non-parametric alternative based on ranges [28]. Statistical significance was considered with a value of *p* < 0.05.

## 3. Results

One hundred twenty-eight (128) treatment episodes were assessed, corresponding to 125 HCV/HIV co-infected patients who had received DAA treatment against HCV with interferon-free regimens in our center from 2015 to 2019. Three patients required re-treatment with DAA for different reasons: one patient due to early discontinuation and two patients due to recurrence (GT-1a and GT-3, respectively), remaining later on SVR12 (Figure 1). Two patients died post-treatment with DAA. One was due to upper gastrointestinal bleeding secondary to bulbar ulcers, anticoagulation, and deep vein thrombosis. Another was due to cerebral hemorrhage after finishing DAA therapy. Only patients who achieved SVR12 were analyzed (n = 123). Three patients who failed and two who died were excluded from the analysis (n = 5). The two deceased patients were undetectable at the end of treatment (EOT), but SVR12 could not be verified, so finally, five patients were excluded, analyzing the remaining 123 patients who achieved SVR12. Concerning HIV, 93% of patients had undetectable plasma viral load (<50 HIV-RNA copies/mL) at baseline.

A total of 69.9% of the SVR12 patients were male and 30.1% female. At the start of treatment, the mean age was 51.3 ± SD 6.7 years. In 87.0% of the patients, a history of intravenous drug use was recorded. A total of 19.5% of the patients were on opioid substitution therapy, methadone being the most frequent (92%). At baseline, 1.6% had consumed an intravenous drug in the previous year, and 4.1% by inhalation or oral route. Cannabis was the most used substance (79.3%), followed by intranasal cocaine (10.3%). In our cohort, 3.4% of the patients had chemsex experience.

The most frequent HCV GT was GT-1. GT-1a: 27.6%, GT-1b: 9.8% and GT-1 other 27.6%. Seventeen-point one percent (17.1%) corresponded to genotype 3 (GT-3), 17.1% to genotype 4 (GT-4) and 1 patient (0.8%) genotype 2 (GT-2).

Regarding accompanying ART during DAA therapy, the use of non-nucleoside analogues (NNRTI) with a regimen based on rilpivirine (RPV) was found in 23.6% of our cohort. It was followed by triple therapy with integrase inhibitor (INI) based on dolute-gravir/abacavir/lamivudine (DTG/ABC/3TC) by 16.3%. The baseline demographics, treatments, HCV genotype and clinical features are shown in Table 1.

The most widely used DAA treatment regimen was sofosbuvir + ledipasvir ± ribavirin (SOF + LEDV ± RBV) in 27.6% of the patients. Followed by paritaprevir/r, ombitasvir and dasabuvir ± ribavirin (3D ± RBV) in 20.3% of the cohort. Among the pan-genotypic DAA, sofosbuvir/velpatasvir (SOF/VEL) stands out in 12.2% and glecaprevir/pibrentasvir (G/P) in 4.9% of the patients. According to the therapeutic targets, 48.8% of the patients received treatments based on NS3/NS4A protease inhibitors and 51.2% NS5A, NS5B inhibitors, as shown in Table 2.

No differences were found in baseline vs. SVR12 comparison in terms of weight (69.2 ± SD 14.7 kg vs. 70.14 ± SD 14.3 kg (*p* = 0.317)) or BMI (23.9 ± SD 4.0 kg/m^2^ vs. 24.7 ± SD 5.6 kg/m^2^ (*p* = 0.165)). Significant differences were found in the measure of total cholesterol (161.3 ± SD 41.0 mg/dL vs. 183.3 ± SD 41.6 mg/dL (*p* < 0.01)) and LDLc (84.6 ± SD 35.0 mg/dL vs. 108.6 ± SD 35.1 mg/dL (*p* < 0.01)).

In the study of liver data (basics laboratory parameters and TE) such as alanine aminotransferase (ALT), baseline mean values of 58.2 ± SD 34.0 U/L and in SVR12 of 22.0 ± SD 16.0 U/L were observed (*p* < 0.01). In albumin values, observing before-after mean values of 4.2 ± SD 0.4 g/dL vs. 4.3 ± SD 0.3 g/dL (*p* < 0.01). Statistically significant differences were also found in baseline vs. SVR12 bilirubin values (0.76 ± SD 0.62 mg/dL vs. 0.65 ± SD 0.53 mg/dL (*p* < 0.05)). Regarding the CTP score, statistically significant differences were found before-after treatment. At baseline, 116 patients (94.3%) were in class A, 7 patients (5.7%) in class B and after achieving SVR12: 121 patients were in class A (98.3%) and 2 patients (1.7%) in class B. CTP mean score was 5.2 ± SD 0.6 points, vs. 5.1 ± SD 0.3 points (*p* < 0.01), as shown in Table 3.

**Figure 2:**
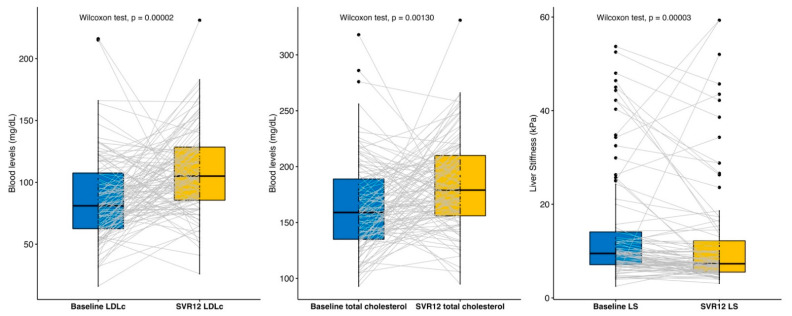
Lipid (LDLc and total cholesterol) and liver stiffness changes baseline versus SVR12. LS: liver stiffness, SVR12: sustained virological response at 12 weeks after treatment, LDLc: low-density cholesterol.

TE’s mean baseline LS value was 13.7 ± SD 13.3 kPa, and after achieving SVR12, a mean value of 11.8 ± SD 12.1 kPa (*p* < 0.01). As shown in Figure 2, using a box-and-whisker plot for paired data [29], we represent the changes that occurred in the baseline cohort versus SVR12, being able to objectify the changes experienced in each episode concerning LDLc, total cholesterol and liver stiffness before-after DAA therapy.

**Figure 3:**
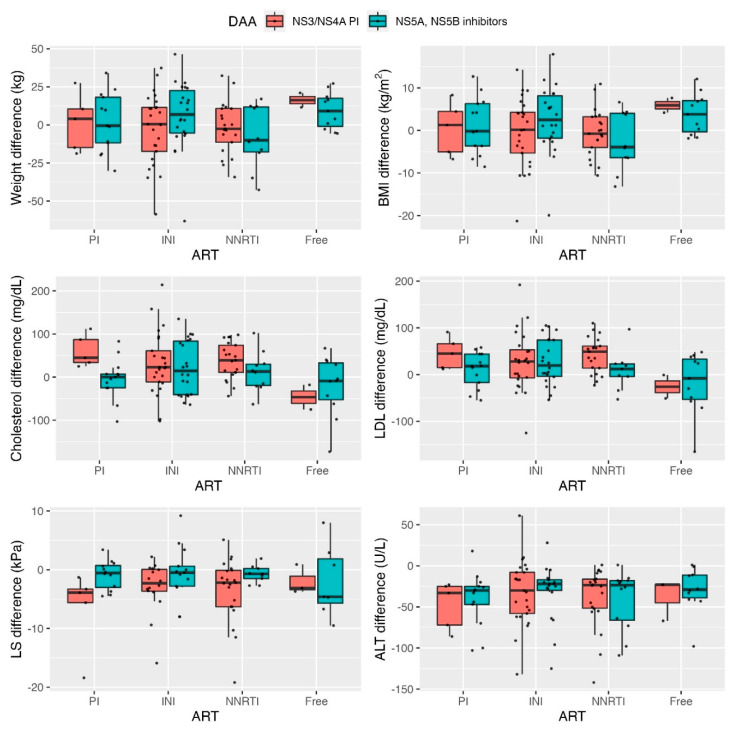
Differences (baseline vs. SVR12) post DAA therapy of the variables of interest in the types of treatments (ART and DAA). DAA, direct-acting antiviral; NS3/NS4 PI, non-structural protein 3/non-structural protein 4A protease inhibitors; NS5A, NS5B; non-structural protein 5A, non-structural protein 5B inhibitors; ART, antiretroviral treatment; PI, protease inhibitor; INI, integrase inhibitor; NNRTI, non-nucleoside reverse transcriptase inhibitor; Free, nucleoside analogue-free therapy. Rank and pseudo-rank hypothesis tests: Weight difference (kg) *p* = 0.3036; BMI difference (kg/m^2^) *p* = 0.3988; Cholesterol difference (mg/dL) *p* = 0.0050; LDL difference (mg/dL) *p* = 0.1180; LS difference (kPa) *p* = 0.4118; ALT difference (U/L) *p* = 0.9126.

Regarding the influence of the interaction between the types of treatments (ART and DAA) used simultaneously in our cohort of patients living with HIV co-infected with HCV, after applying rank and pseudo-rank hypothesis tests, no significant differences were found concerning weight or BMI (*p* > 0.05). Analyzing the lipid profile (total cholesterol and LDLc), a synergy has been confirmed between the use of ART based on PI (ATV or DRV) and the use of DAA based on NS3/NS4A PI (*p* = 0.0050) in terms of the global increase of cholesterol total. No significant interactions in the combined use of ART and DAA in LDLc (*p* = 0.1180) were observed. Significant differences neither observed between the different types of ART and DAA combinations in terms of ALT and LS. An overall improvement was observed without influence or synergy between the different therapy combinations (*p* > 0.05). As shown in Figure 3, it is possible to objectify the evolution of the magnitudes of the differences depending on the combinations of treatments used.

**Figure 4:**
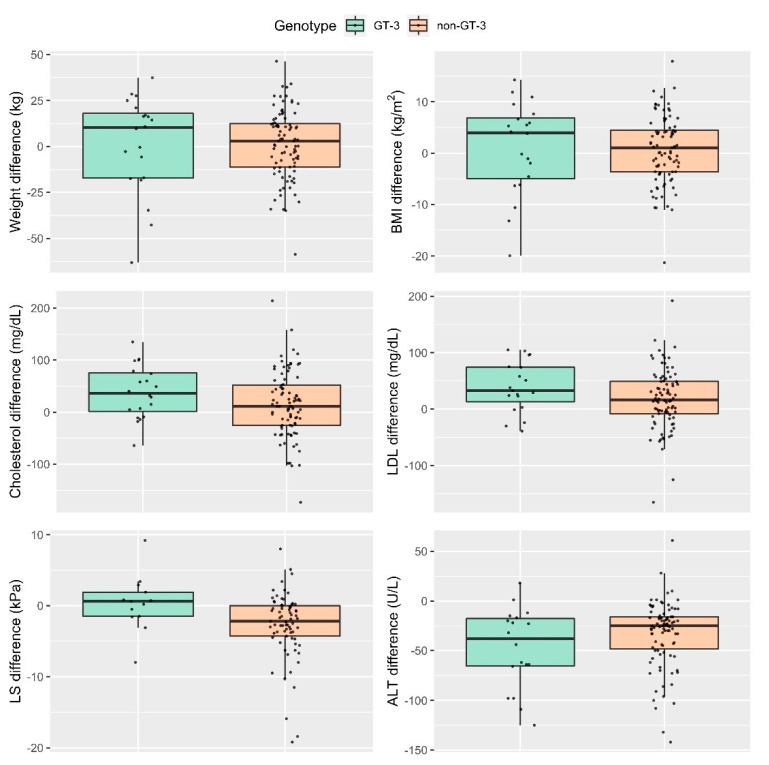
Differences (baseline vs. SVR12) post DAA therapy of the variables of interest in GT-3 vs. non-GT-3. Wilcoxon test: Weight difference (kg) *p* = 0.57; BMI difference (kg/m^2^) *p* = 0.51; Cholesterol difference (mg/dL) *p* = 0.099; LDL difference (mg/dL) *p* = 0.085; LS difference (kPa) *p* = 0.011; ALT difference (U/L) *p* = 0.25.

Assessing the magnitude of the differences after facing GT-3 vs. others in anthropometric (weight and BMI), lipid (total cholesterol, LDLc) and liver (ALT and LS) changes after achieving SVR12, no significant differences have been obtained in anthropometric, lipid and ALT changes (*p* > 0.05). Patients with non-GT-3 have presented a greater magnitude of differences (baseline vs. SVR12) with an improvement in LS vs. GT-3 (non-GT-3: −3.2 ± SD 13.3 vs. GT-3: −1.1 ± SD 6.8 kPa, *p* < 0.05), as shown in Figure 4.

## 4. Discussion

This study assessed the early metabolic impact of achieving SVR12 against HCV after DAA therapy in terms of anthropometric (weight, body mass index), lipid profile, and liver (basic necroinflammatory laboratory parameters and transient elastography) changes in HCV/HIV co-infected patients.

Regarding the referred anthropometric variables (weight and BMI), there has been no significant weight gain after achieving SVR12. This fact is consistent with other studies such as Iossa et al., where they did not observe relevant short-term anthropometric changes after DAA therapy [13]. Although the upward trend in weight and BMI in the short term has been evidenced, it could represent a clinically relevant fact in the long term if maintained over time.

In accordance with previous observational studies reports in patients with HCV chronic infection [13], our study seems LDLc and total cholesterol increase after DAA therapy in parallel to favourable changes in basic parameters of function and LS after being successfully treated against HCV with DAA therapy. The benefit can be seen early in the short-term (12 weeks of follow up). The relevance of our findings lies in the fact that they occur in a cohort of PLWHIV co-infected with HCV, where data are limited. It can also confirm the results in other cohorts such as ICONA and HepaICONA foundation Cohort Study by Spaziante et al. They show an improvement in liver function after successful DAA therapy associated with an increase in serum lipids levels after HCV eradication that should be monitored [21]. Our study, therefore, confirms the rise in total cholesterol and LDLc levels after HCV clearance, the early regression in liver function parameters and LS also in HCV/HIV co-infected patients implying a relevant finding for routine clinical practice.

Our cohort’s baseline lipid mean values showed lower serum levels of total cholesterol and LDLc at baseline compared to achieving SVR12. This can be explained by the chronic HCV infection at baseline since HCV modulates intrahepatic cholesterol biosynthetic pathways to promote viral replication [15]. HCV uses the lipid metabolic machinery of the infected host for its own viral proliferation and replication, interacting with lipid droplets to form the lipid-rich virus capsid and complete its replicative cycle [30]. After HCV SVR or cure, our cohort experienced a mean increase of the lipid value concerning the baseline situation before the introduction of DAA. Thus, the eradication of HCV could worsen the lipid profile by removing its lipid-lowering influence and an individual predisposition of the patient that was previously counteracted by the HCV infection [16,31]. Future research may allow us to draw specific conclusions about metabolic changes and check if the preliminary negative impact on the lipid profile, by reducing the lipid-lowering effect induced by HCV, could be seen long-term offset by optimizing glycemic-hydrocarbon metabolism with lower insulin resistance. It could also check the impact on metabolic disorders (e.g., diabetes) since HCV may be more detrimental to energy homeostasis than a mere elevation of cholesterol. This would lead to less subclinical atherosclerosis that seems to improve after HCV cure [32,33].

The HCV-fibrosis interaction is a dynamic process. A decrease in necro-inflammatory changes and improved LS are expected after DAA therapy. We observed a global improvement in the cohort regarding basic function liver parameters and LS comparing baseline versus SVR12. Our findings were consistent with what has been described in the literature in other cohorts, such as Pietsch et al., whose study allows assessing rapid fall in LS over the sort-term course, driven by declines in necroinflammation liver parameters [34,35]. Thus, more research regarding these long-term findings could help reduce the risk of progression and development of hepatocellular carcinoma in PLWHIV with HCV co-infection.

Delving into the magnitude of the differences concerning the treatments used, contemplating the use of ART and DAA together, we show how, in general terms, there is no significant interaction between both groups. Thus, all of the combinations used behave similarly or have had the same impact. The exception was the combination of HIV protease inhibitors used, which, in conjunction with NS3/NS4A PI, have shown a short-term synergistic effect in raising total cholesterol but not significant in LDLc. This is in line with what is described in the literature such as Lee et al. ritonavir representing an example of PI that motivates these lipid metabolism disorders [36]. So, this finding may imply tighter monitoring when both protease inhibitors are combined.

Authors such as Abenavoli et al. have postulated that liver steatosis is more frequent in patients infected with the HCV-GT-3 due to the direct effect of viral proteins with a greater possibility of metabolic disorders. In this sense, it has been compared whether the anthropometric, lipid and liver changes results obtained in our study have been influenced by the viral GT, comparing GT-3 vs. non-GT-3. Patients with GT-3 have experienced a baseline LS significative improvement vs. SVR12 LS of lesser magnitude than patients infected by the rest of the GTs. These results should be interpreted with caution since patients infected with GT-3 could present a higher liver steatosis baseline degree due to being infected with HCV-GT-3 and the short-term evaluation changes in our research [37]. The presence of liver steatosis can cause an overestimation of the LS values measured by TE, so an adequate correlation by controlled attenuation parameter (CAP) or an ultrasound (US) evaluation could control it [38].

This study has several limitations. One of the most important is having received treatments during 2015–2016 when the use of DAA was prioritized in patients with advanced fibrosis. This center is a reference center for a province with 727,945 inhabitants. During this period, all HIV/HCV co-infected patients from the province of Almeria were treated at our center. This aspect should be considered when assessing the external validity of our findings. Another limitation of our research is that it did not have a control group due to the own design of the study. DAA therapy was assessed according to the current recommendations of the clinical practice guidelines and health authorities. In the beginning, pan-genotypic DAA therapy was not available since they were not marketed. We were only allowed to treat patients with advanced fibrosis as in the rest of the country. Another limitation is that the evaluation performed is short-term. This aspect could have influenced some results, such as the findings related to weight and BMI, in which no significant statistical differences were found. Long-term studies with a follow-up of one or two years may be helpful to clarify if the changes concerning lipid parameters are stable along the time. It has not been possible to provide the CAP value or US assessment since they were not collected in the study despite being data from routine practice, and it could have been of interest. Data about glycemia, Homa-IR index, and history of concurrent comorbidities were not collected due to the design of the study protocol. The analysis of these data could be considered for future research. Finally, it is important to consider that the size of our sample could limit the external validity of our findings. Albeit it could be helpful to perform this research with a bigger sample, we must also consider that we obtained statistically significant results that can be relevant for actual clinical practice. There are also other similar studies published with similar sample sizes [20,39].

Our research also had some strengths. The most important is that this study helps to acknowledge the situation and behavior of a cohort of PLWHIV with HCV co-infection in real life. We must also consider that significant metabolic changes have been evident early on, without detriment to the main goal of the interferon-free DAA therapy, which is the cure of HCV. It is also important to consider that we have studied a real-life population, an aspect that has been important to obtain some significant results. Due to these aspects mentioned above, our findings can be relevant for actual clinical practice. Our research has delved into the differences based on the synergy of interaction between each treatment family used. It will allow designing a predictive model where TE can play an important role in the follow-up of these patients.

## 5. Conclusions

The use of direct-acting antivirals against HCV in HCV/HIV co-infected patients implies a significant early increase of the lipid profile derived from the cure of Hepatitis C, requiring close clinical follow-up with individually therapeutic decision-making with special vigilance when combining protease inhibitors. The eradication of HCV in PLWHIV implies that, in the early stages, global improvements in liver function data and LS regardless of the combination of ART and DAA used. This could help reduce the risk of progression and development of hepatocellular carcinoma. Therefore, these findings can be relevant for actual clinical practice and future research. HCV-GT-3/HIV co-infection could require closer follow-up in terms of residual fibrosis. Well-designed studies exploring the development of long-term metabolic disorders in HCV-GT-3/HIV co-infected patients could be helpful.

## Figures and Tables

**Table 1:** Baseline characteristics of SVR12 patients based on age, sex, transmission route, opioid substitution therapy, ART, DAA, and HCV genotype (n = 123) *.

n = 123	Mean	SD
Age (years)	51.3	±6.7
Sex	n	%
Male	86	69.9
Female	37	30.1
HCV/HIV transmission route	n	%
IDU	107	87.0
HTX	10	8.1
MSM	4	3.3
Other (blood transfusion)	2	1.6
Opioid substitution therapy (OST)	n	%
no	99	80.5
yes	24	19.5
Baseline Child-Turcotte-Pugh (CTP) score	n	%
A	116	94.3
B	7	5.7
C	0	0
HCV Genotype (GT)	n	%
GT-1a	34	27.6
GT-1b	12	9.8
GT-1-other	34	27.6
GT-2	1	0.8
GT-3	21	17.1
GT-4	21	17.1
DAA combination	n	%
SOF/DCV ± RBV	14	11.4
SOF/SMV ± RBV	21	17.1
3D ± RBV	25	20.3
2D ± RBV	3	2.4
SOF/LEDV ± RBV	34	27.6
GZP/ELB	5	4.1
SOF/VEL	15	12.2
G/P	6	4.9
ART combination	n	%
ABC/3TC + ATV	2	1.6
ABC/3TC + DTG	20	16.3
ABC/3TC + RAL	2	1.6
ATV + RAL	1	0.8
TDF/FTC + ATV	3	2.4
DRV	2	1.6
DRV+3TC, INI or MVC	7	5.7
TDF/FTC/RPV	29	23.6
TDF/FTC + RAL	16	13.0
EVG/c/FTC/TDF	5	4.1
DRV/b+FTC/TDF	3	2.4
FTC/TDF + DTG	5	4.1
FTC/TDF/ EFV	3	2.4
Other	25	20.3

* SOF, sofosbuvir; DCV, daclatasvir; RBV, ribavirin; 3D, paritaprevir/r, ombitasvir, and dasabuvir; 2D, paritaprevir/r and ombitasvir; LEDV, ledipasvir; GZP, grazoprevir; ELB, elbasvir; G/P, glecaprevir co-formulated with pibrentasvir; SMV, simeprevir; ABC, abacavir; 3TC, lamivudine; ATV, atazanavir; RAL, raltegravir; TDF, tenofovir dimethyl fumarate; FTC, emtricitabine; DRV, darunavir; DRV/p, boosted darunavir (cobicistat or ritonavir); MVC, maraviroc; RPV, rilpivirine; EVG/c, elvitegravir/cobicistat; IDU, intravenous drug use; HTX, heterosexual; MSM, men who had sex with men; child-Turcotte-Pugh (CTP) score: A (5–6 points), B (7–9 points), C (10–15 points); GT, HCV genotype. DAA, direct-acting antiviral; ART, antiretroviral treatment.

**Table 2:** Contingency table with frequencies n (%) of ART and DAA use *. χ²: 11.4438, *p* = 0.0096.

	NS3/NS4A PI	NS5A, NS5B Inh.	Total
PI	6 (4.9)	13 (10.6)	19 (15.4)
INI	28 (22.8)	26 (21.1)	54 (43.9)
NNRTI	23 (18.7)	12 (9.8)	35 (28.5)
Free	3 (2.4)	12 (9.8)	15 (12.2)
Total	60 (48.8)	63 (51.2)	123 (100.0)

* PI, protease inhibitor; INI, integrase inhibitor; NNRTI, non-nucleoside reverse transcriptase inhibitor; Free, nucleoside analogue-free therapy; NS3/NS4A PI, non-structural protein 3/non-structural protein 4A protease inhibitors; NS5A, NS5B inh.; non-structural protein 5A, non-structural protein 5B inhibitors.

**Table 3:** Before-after laboratory value and anthropometric parameters *.

Baseline Variables	Mean	SD	SVR12 Variables	Mean	SD	*p*-Value
Baseline weight	69.2	14.7	SVR12 weight	70.4	14.3	*p* = 0.317
Baseline BMI	23.9	4.0	SVR12 BMI	24.7	5.6	*p* = 0.165
Baseline cholesterol	161.3	41.0	SVR12 cholesterol	183.3	41.6	*p* < 0.01
Baseline LDLc	84.6	35.0	SVR12 LDLc	108.6	35.1	*p* < 0.01
Baseline ALT	58.2	34.0	SVR12 ALT	22.0	16.0	*p* < 0.01
Baseline albumin	4.2	0.4	SVR12 albumin	4.3	0.3	*p* < 0.01
Baseline bilirubin	0.8	0.6	SVR12 bilirubin	0.6	0.5	*p* < 0.05
Baseline CTP score	5.2	0.6	SVR12 CTP score	5.1	0.3	*p* < 0.01
Baseline TE	13.7	13.3	SVR12 TE	11.8	12.1	*p* < 0.01

* Weight (Kg); BMI, body mass index (weight in kg / height in m^2^); SVR12, sustained viral response 12 weeks after completing DAA treatment. Cholesterol (mg/dL), LDLc (mg/dL); ALT (U/L); albumin (g/dL); bilirubin (mg/dL); CTP score: Child-Turcotte-Pugh score (points); Transient elastography (TE) (kPa). *p*-value after applying Wilcoxon before-after test to each variable: not significant (*p* > 0.05); statistical significance (*p* < 0.05).

## Data Availability

All data are contained within the article.
